# STRUCTURE-FUNCTION RELATIONSHIPS OF MUCOCILIARY CLEARANCE IN HUMAN AIRWAYS

**DOI:** 10.21203/rs.3.rs-4164522/v1

**Published:** 2024-04-25

**Authors:** Doris Roth, Ayşe Tuğçe Şahin, Feng Ling, Christiana N. Senger, Erik J. Quiroz, Ben A. Calvert, Anne M. van der Does, Tankut G. Güney, Niels Tepho, Sarah Glasl, Annemarie van Schadewijk, Laura von Schledorn, Ruth Olmer, Eva Kanso, Janna C. Nawroth, Amy L. Ryan

**Affiliations:** 1 Helmholtz Pioneer Campus, Institute of Biological and Medical Imaging, and Member of the German Lung Research Center (DZL CPC-M), Helmholtz Zentrum München, Neuherberg, D-85764, Germany; 2 Chair of Biological Imaging at the Central Institute for Translational Cancer Research (TranslaTUM), School of Medicine, Technical University of Munich, Munich, D-81675, Germany; 3 Hastings Center for Pulmonary Research, Division of Pulmonary, Critical Care and Sleep Medicine, Department of Medicine, University of Southern California, Los Angeles, CA 90033; 4 Department of Aerospace and Mechanical Engineering, University of Southern California, Los Angeles, CA 90089, USA; 5 Department of Stem Cell Biology and Regenerative Medicine, University of Southern California, Los Angeles, CA 90033, USA; 6 PulmoScience Lab, Department of Pulmonology, Leiden University Medical Center, Leiden, the Netherlands; 7 Leibniz Research Laboratories for Biotechnology and Artificial Organs (LEBAO), Department of Cardiothoracic, Transplantation and Vascular Surgery (HTTG), Hannover Medical School, Hannover, D-30625, Germany; 8 Biomedical Research in End stage and Obstructive Lung Disease (BREATH), Member of the German Center for Lung Research (DZL), Hannover Medical School, Hannover, D-30625, Germany; 9 REBIRTH-Research Center for Translational and Regenerative Medicine, Hannover Medical School, Hannover, D-30625, Germany.; 10 Department of Anatomy and Cell Biology, Carver College of Medicine, University of Iowa, IA 52242, USA

## Abstract

Our study focuses on the intricate connection between tissue-level organization and ciliated organ function in humans, particularly in understanding the morphological organization of airways and their role in mucociliary clearance. Mucociliary clearance is a key mechanical defense mechanism of human airways, and clearance failure is associated with many respiratory diseases, including chronic obstructive pulmonary disease (COPD) and asthma. While single-cell transcriptomics have unveiled the cellular complexity of the human airway epithelium, our understanding of the mechanics that link epithelial structure to clearance function mainly stem from animal models. This reliance on animal data limits crucial insights into human airway barrier function and hampers the human-relevant *in vitro* modeling of airway diseases. This study, for the first time, maps the distribution of ciliated and secretory cell types along the airway tree in both rats and humans, noting species-specific differences in ciliary function and elucidates structural parameters of airway epithelia that predict clearance function in both native and *in vitro* tissues alike. By uncovering how tissue organization influences ciliary function, we can better understand disruptions in mucociliary clearance, which could have implications for various ciliated organs beyond the airways.

## INTRODUCTION

Mucociliary clearance (MCC) is a critical mechanical barrier mechanism of the human airways^1–3^. In MCC, the beating of specialized multiciliated cells propel a layer of mucus across the epithelial surface, effectively trapping and removing inhaled particles and pathogens^4,5^. The mucus is produced by the submucosal glands and a variety of secretory cells interspersed among ciliated cells. These secretory cells include mucin-secreting goblet cells, club cells and their intermediate stages^6^. The composition of secretory cell types has a profound impact on the rheology and flowability of mucus^7–9^. Failure of MCC contributes to the debilitating pathophysiology of many respiratory diseases, including chronic obstructive pulmonary disease (COPD), primary ciliary dyskinesia, asthma, and cystic fibrosis^10,11^. However, our understanding of how diseases impair MCC remains limited by an incomplete knowledge of the mechanics that link secretory and ciliated cell organization to MCC in humans^3^ since most *in vivo* studies rely on animal models^12–14^. Obtaining data on live ciliary beat and clearance function in human tissue poses challenges and remains exceedingly rare in the literature^15,16^. Furthermore, the organization of secretory and ciliated cells at the epithelial surface is underexplored, as conventional histology typically reveals cross-sectional rather than surface-lining tissue architecture. While providing important insights about cellular heterogeneity, transcriptional profiling commonly does not resolve spatial organization^17,18^ and may not accurately reflect protein expression^19^.

The lack of such crucial data limits not only our understanding and capability to model human MCC but may also undermine correct diagnosis and early intervention in disease. For instance, a recent study revealed that while luminal surface ciliation in the mouse trachea is relatively low (approximately 40%), the specific distribution and orientation of ciliary beat ensure robust and efficient particle clearance^14^. However, the authors could only speculate whether these design principles directly translate to humans, where such quantitative insights could help refine treatments for conditions impairing ciliary beat, such as PCD^20^, or causing loss of ciliated cells, such as asthma^21^. The limited human-relevancy of animal studies has spurred the development of innovative *in vitro* human airway epithelial models^22–24^. Nevertheless, without quantitative benchmarks of MCC in humans, the ability of these models to accurately represent human physiology remains unproven. Furthermore, the proportions of ciliated and various secretory cell types are believed to vary along the airway tree within and between humans and rats^25^. It remains unclear whether this heterogeneity results in species-specific regional differences in MCC, which is pertinent for studying diseases that alter the regional proportions of airway epithelial cell types^26^.

To address these gaps, we have established a quantitative map of the distribution of ciliated, club, and goblet cells along the human and rat airway tree’s surface lining. We have evaluated species-specific differences in the associated particle clearance and utilized these data to develop quantitative metrics and physics-based computational models capturing key MCC characteristics in lower airway epithelia. Finally, we deploy our comprehensive quantitative framework to benchmark the structural and functional properties of a variety of *in vitro* human airway epithelial culture conditions against native human airway epithelia. The data presented significantly enhances our understanding of MCC mechanics and will improve the translational potential of *in vitro* models for studying respiratory diseases and developing therapeutic interventions.

## RESULTS

### Tracheo-bronchial ciliation is higher in human airways compared to rat.

**Figure 1:**
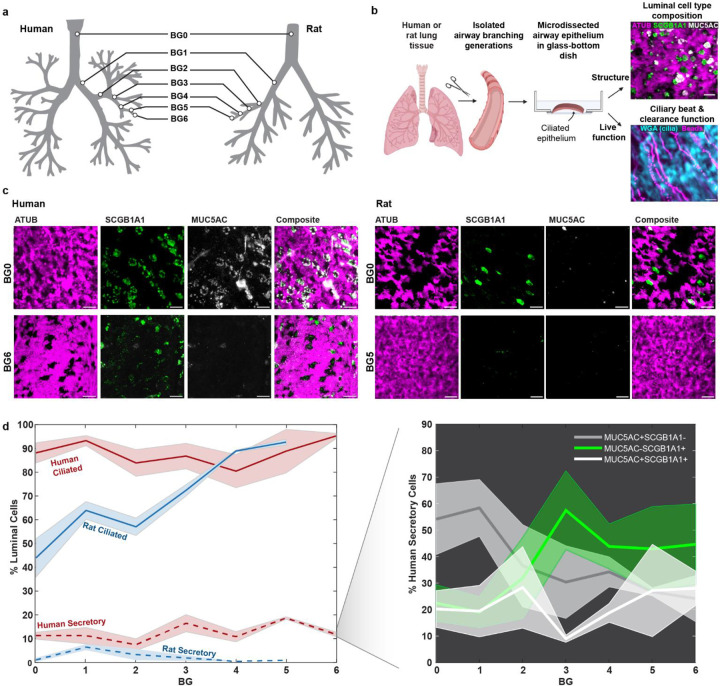
Luminal epithelial cell type composition differs along the human and rat airway tree. a, Airway branching generations in human and rat investigated in this study included BG0–6 in humans and BG0–5 in rats. b, Workflow for imaging luminal epithelial cell type composition and ciliary beat and clearance function in airway samples. c Example IF staining of cilia (ATUB, magenta) and secretory cells (SCGB1A1, green; MUC5AC, grey) in human and rat airway epithelium in BG0 (trachea) and BG5/6. Scale bar: 20 μm. D, Quantification of luminal cell proportions labeled with ATUB (ciliated cells) or with MUC5AC and/or SCGB1A1 (secretory cells) as a function of airway branching generation in human and rat airway epithelium. Inset: Percentage of human secretory cell population positive for only MUC5AC (grey), only for SCGB1A1 (green), or for both (white) as a function of branching generation. Solid line: mean, shaded region: SEM. For donor numbers and information see **Supplementary Tables S1 & S2**.

Live ciliary beat and particle clearance^15,27^ was measured in freshly isolated airway epithelial tissue from respiratory tree branching generation (BG)0 through BG6 derived from 3 human whole lungs with no prior history of chronic lung disease, and BG0 through BG5 from healthy rat whole lungs (Fig. 1a; for detailed donor information and numbers see **Supplementary Tables S1 & S2**). The human BG6 samples were at, or below, 2 mm in diameter (**Supplementary Fig. S1**) and therefore constituted the transition to small airway morphology^28^. While such a definition of small airways does not exist for rodents, we covered a comparable fraction of the conducting airways along the proximal-distal axis in both systems (7 BGs of 16 BGs in humans^29^, and 6 BGs of 14 BGs in rats^30^). Subsequently, we quantified the luminal (i.e., surface-lining) cell type composition in these samples and, to increase donor numbers, in additional fixed human bronchial rings. The percentage of cells at the mucosal surface expressing cell-specific markers was quantified using mucin 5AC (MUC5AC) to mark goblet cells, secretoglobin family 1A member (SCGB1A1) to label club cells, and acetylated alpha-tubulin (ATUB) to stain ciliated cells^4^ (Fig. 1b–c). Luminal cell boundaries were revealed by F-actin staining of the cytoskeleton, allowing total cell numbers and percentages of cells labeled with each specific marker to be determined (See Methods and **Supplementary Figs. S2 & S3**). The results are summarized in Fig. 1d, for details see **Supplementary Tables S3 & S5.** Intriguingly, human airways exhibited a robustly high ciliated cell proportion, or cilia coverage, of 88 ± 2% (mean±SEM) across all analyzed BGs, whereas cilia coverage in rat airways gradually increased along the airway tree, ranging from 44 ± 8% in trachea to 92 ± 1% in BG5. This contrasted with results obtained from classic histology or gene expression, which has suggested a lower ciliation level of 30–50% in human trachea and increasing ciliation in higher BGs^25,31–34^ (**Supplementary Fig. S4**). On the other hand, our analysis of secretory cell levels matched trends reported in these studies. In all examined human BGs, 10–20% of luminal cells were positive for either MUC5AC, SCGB1A1, or both. Within this secretory cell population, the proportion of MUC5AC+ SCGB1A1− (goblet) and MUC5AC− SCGB1A1+ (club) cells varied as a function of BG (Fig. 1d, inset). Goblet cells dominated the trachea bronchial region whereas club cells became increasingly frequent in BG3 and higher. On average, 15 and 30% of the labeled secretory population were MUC5AC+ SCGB1A1+ (double-positive hybrid/transitionary) cells, consistent with transcriptional and histological studies^31,35,36^ (for additional staining see **Supplementary Fig. S5A & B**). A comparable analysis was inconclusive for the rat secretory cells due to the variable, but overall low (<10%), abundance of cells positive for MUC5AC and/or SCGB1A1, which matches literature^25^ (for additional stainings see **Supplementary Fig. S5C**). Since rat airways are thought to contain a high abundance of so-called “serous” secretory cells^25^ for which no protein markers have been reported, additional secretory cells could be present.

### Human airways achieve greater clearance per ciliary beat than rat airways.

**Figure 2:**
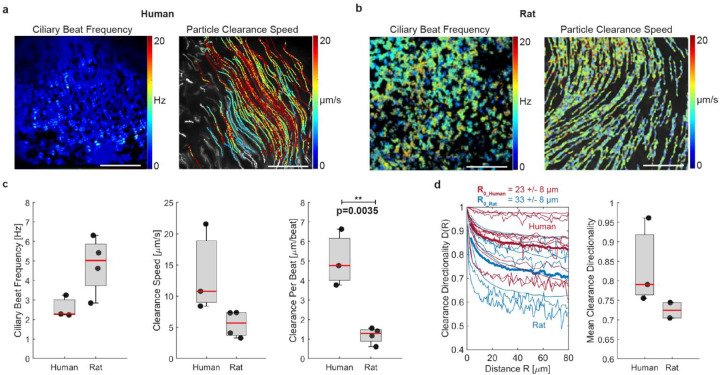
Ciliary beat and particle clearance function differ between human and rat airway. a, Representative measurement of ciliary beat frequency (CBF) and associated particle clearance trajectories and speed in a human airway epithelial sample (BG2). b, Same measurements in rat airway epithelial sample (BG1). c, Quantification of average CBF, particle clearance speed, and clearance per beat (CPB) in human airways BG0–6 and rat airways BG0–1. d, left: Clearance directionality as a function of distance in human (red) and rat (blue) airways. Thick lines are average curves. Right: Mean directionality over a flow distance of 80 μm. Boxplots: Each solid dot is the mean value of one donor (across multiple BGs); red line is median of distribution; significance was assessed with Kruskal–Wallis test.

The differences in cilia coverage between species suggested differences in particle clearance, which we proceeded to explore by measuring live ciliary beat and fluorescent bead transport in the densely ciliated human airways (BG0 to BG6) compared to the sparsely ciliated rat airways (BG0 and BG1) (Fig. 2a–b, **Supplementary Videos S1 & S2)**. These regions correspond to the trachea, main stem bronchi, and bronchial regions in humans and rats. Ciliary beat frequency (CBF) recorded at room temperature was 2.9 ± 0.5 Hz human tissue and 4.7 ± 1.7 Hz in rat tissue. Associated particle clearance speed reached 11.6 ± 5.9 μm/s in humans and 5.6 ± 2.8 μm/s in rats. To compare the level of particle clearance despite the differences in CBF, we derived the normalized “clearance speed per beat” (CPB) by dividing the average clearance speed by the average CBF for each field of view. CPB measures how far a particle is transported per ciliary beat cycle and has the units μm/beat. This analysis revealed a significantly higher CPB in human tissue (4.2 ± 2.5 μm/beat) compared to rat tissue (1.2 ± 0.5 μm/beat) (Fig. 2c). We also investigated particle clearance directionality D as a function of distance R, defined as $D(R)=\frac{\left|\langle v\rangle \right|}{\left\langle |v|\right\rangle }$,where ⟨⟩ indicates the mean, ∥ indicates the magnitude, and v indicates flow speed. D(R) ranges from 0 to 1, where a number near zero indicates highly convoluted flow and a number near 1 indicates straight and unidirectional flow, and it decays with increasing distance. Each trace was fitted with a decaying exponential, $D(R)e^{\frac{-R}{R_{0}}}$, to estimate the correlation length $R_{0}$. This analysis revealed a similar characteristic length scale of the correlated flow in human $\left(R_{0}=23\pm 8\;\mu m\right)$ and rat airways $\left(R_{0}=33\pm 8\;\mu m\right)$ (Fig. 2d, left). We also compared mean particle clearance directionality over multiple cell lengths at R=80μm, which was higher in human compared to rat tissues, indicating straighter transport (Fig. 2d, right). However, since *ex vivo* airways are naturally concave, the imaging plane sometimes diverted from the surface, such that particle trajectories appeared distorted (**Supplementary Fig. S6A**); therefore, the data was noisy, and the difference did not reach significance. All measurements were completed in tissues from healthy, adult lungs that were washed, mucus-free and submerged in aqueous saline buffer, suggesting that the differences in CPB and clearance directionality were due to species-specific properties of ciliary beat and organization^14^ rather than defective^37^ or immature ciliary beat^16^, or altered mucus properties^38,39^.

**Figure 3:**
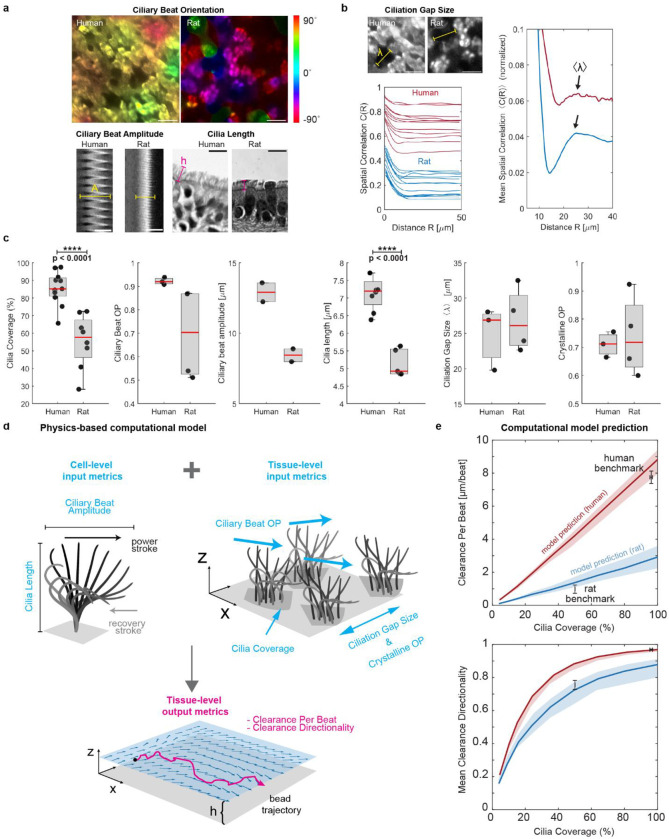
Quantitative analysis of ciliary beat parameters and their impact on clearance function. a, Cell-level analysis of ciliary beat orientation based on beat trajectories (scalebar: 20 μm), ciliary beat amplitude based on kymograph span (scalebar: 10 μm), and cilia length based on histology sections (scalebar: 10 μm). b, Tissue-level analysis of ciliation gap size *λ* using spatial correlation function *C(R)* of ciliated regions in multiple fields of view (left plot) where the first local maximum of the mean correlation curve reveals mean λ (right plot). Scalebar: 20 μm. c, Average cilia coverage, ciliary beat order, ciliary beat amplitude, cilia length, ciliation gap size and crystalline order parameter measured in human BG0–6 and rat BG0–1. Each solid dot is the mean value of one donor (across multiple BGs), red line is median of distribution; significance was assessed with Kruskal–Wallis test. More donors were available for measurements that could be done in fixed tissue. b, Schematic of ciliary input metrics on cell- and tissue-level used to predict output metrics of tissue-level clearance using physics-based computational model. c, Predicted clearance per beat and clearance directionality in human (red, BG0–6) and rat (blue, BG0–1). Solid line represents mean prediction, shaded area shows uncertainty based on spread of input metrics. Black data points and error bars represent experimental human and rat benchmark data (mean ± SEM) indicating a good match of the model predictions.

To capture these species-to-species differences in ciliary activity, we measured multiple ciliary properties at the single cell and tissue level. On the cellular level, we assessed average ciliary beat orientation, i.e., the angle of the ciliary beat axis, as well as ciliary beat amplitude and cilia length (Fig. 3a). At the tissue-level, we analyzed the spatial distribution of ciliated cells using the spatial correlation function C(R) (see Methods) to find λ, the average gap distance between ciliated areas^14^ (Fig. 3b). We computed the relative variability of λ using the crystalline order parameter (COP), defined as crystalline $OP=1-\frac{\sigma \sqrt{2}}{\left\langle \lambda \right\rangle }$ where σ is the standard deviation of λ between field of views^14^. We also determined the degree of alignment of ciliary beat in each field of view using the director-free orientational order parameter defined as ciliary beat $OP=\sqrt{\langle sin2\theta \rangle^{2}+\langle cos2\theta \rangle^{2}}$, where θ are the measured angles. The ciliary beat OP ranges from 0 to 1, where 0 indicates randomly distributed ciliary beat orientations, and 1 indicates spatial alignment of beat.

Using these metrics, we compared the human airways at BG0–6 with the rat airways at BG0–1. Shown by the proximal-distal cell type analysis (Fig. 1d), coverage with ciliated cells in these regions was significantly higher in the human airways (85.2 ± 2.8%) compared to rat airways (55.4 ± 5.4%) (Fig. 3c). We found that cilia length was significantly higher in human compared to rat tissue (human: 7.1 ± 0.5 μm; rat: 5.1 ± 0.13 μm), consistent with literature^16,40^. Ciliary beat OP and amplitude were also higher in human samples, but, with data from only 2 donors available, the difference did not reach significance. Ciliation gap sizes were similar in human (λ=24.5±2.9μm) and rat airways (λ=26.8±2.2μm) and, notably, were thus comparable to the mean correlation length $R_{0}$ of the cilia-driven flow (23 μm and 33 μm, respectively). This is consistent with prior studies in mice showing that the spatial organization of ciliated cells imprints onto the emergent flow patterns^14^. Crystalline OP was also of similar magnitude between human and rat tissue.

To understand how the functional “output metrics” CPB and clearance directionality emerge from structural “input metrics,” we developed a simple hydrodynamic model inspired by force singularity models of cilia^41–43^ to simulate particle clearance due to cilia submerged in aqueous liquid, similar to our experimental measurement conditions (Fig. 3d, **Supplementary Figs. S7 & S8, Supplementary Methods**). Here, we model each ciliated cell as a single regularized Stokeslet that scales with cilia beat amplitude, points horizontally in the effective stroke direction, and is located at one cilia length above a no-slip wall that represents the stationary cell surface. The position of ciliated cells and the orientation of the corresponding Stokeslets are chosen such that the simulated epithelium conforms to the desired input metrics such as cilia coverage and ciliary beat OP, patchiness, and crystalline order parameter. Next, the resulting fluid velocity field and tracer particles trajectories were computed to derive CPB and clearance directionality as a function of cilia coverage. To validate the model, we confirmed that it correctly predicted the most dependable clearance measurements in human and rat samples, i.e., from recordings with minimal sample warp where particles could be recorded right above the cilia layer, thereby minimizing distance dependent loss of speed (**Supplementary Figure S6B).** In these human recordings, a cilia coverage of 94% resulted in a clearance performance of CPB= 7.75 ± 1 μm/beat and a mean directionality value of 0.97 ± 0.1 (Fig. 3e, data point “human benchmark”). The model further predicted that CPB is linearly dependent on cilia coverage, while clearance directionality is a steeply rising function that exponentially converges to its maximum above a certain coverage fraction (Fig. 3e, red curves). We also computed these curves using the input parameters measured in rat airways. The predicted rat-specific curves (Fig. 3e, blue curves) fall below the human-specific curves because of the lower values in ciliary beat OP, amplitude, and cilia length in rats compared to humans (Fig. 3c). This means that for identical cilia coverage, the maximal CPB and clearance directionality are lower in rats. Intriguingly, the experimentally measured benchmark values of CPB and clearance directionality measured in rats again match the range predicted by the model (Fig. 3e, data point “rat benchmark”), suggesting that despite its simplicity, our model captures key structure-function relationships.

### *In vitro* airway cultures rarely match human mucociliary properties.

**Figure 4:**
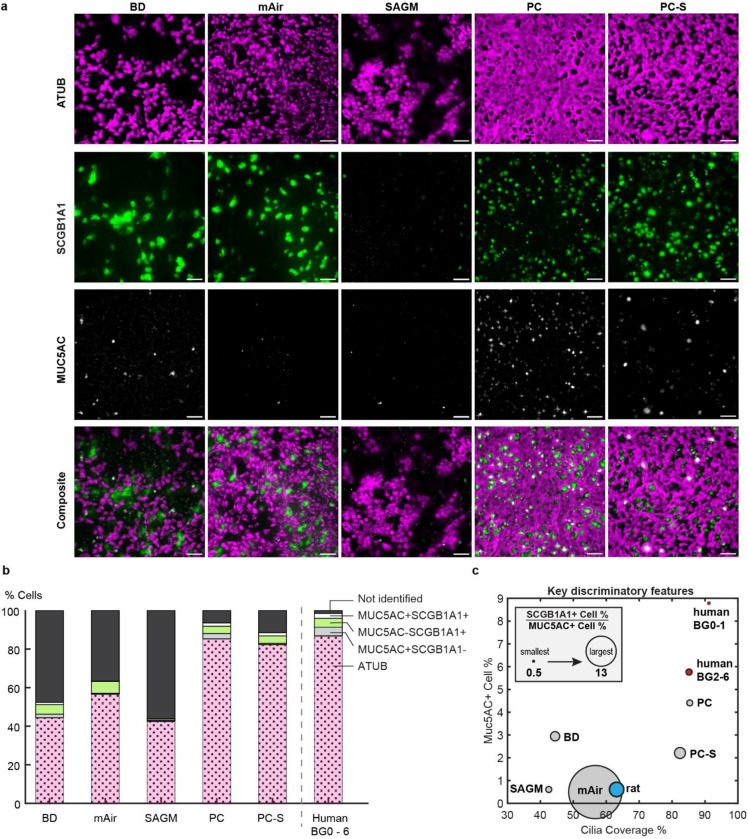
Cell culture media determine *in vitro* luminal cell type composition. a, Representative IF-images of primary human airway epithelial cultures from 1 donor grown in different differentiation media for 28 days at ALI and stained for cilia (ATUB, magenta) and secretory cell markers (SCGB1A1, green; MUC5AC, white). Scalebar, 40 μm. b, Average luminal cell type composition based on IF-staining across N=3–6 donors (**Supplementary Tables S1 & S2**). c, Mapping of IF-staining data of *in vitro* and *ex vivo* samples onto three dimensions. y-axis: percentage of MUC5AC+ cells; x-axis: cilia coverage, i.e., percentage of ciliated (ATUB+) cells; circle diameter: ratio of SCGB1A1+ to MUC5AC+ cell percentages.

We next applied our structural and functional metrics to assess differentiated air-liquid interface (ALI) cultures of primary human airway epithelial cells. ALI cultures are typically used to study human airway diseases and hence there is great interest in establishing human airway-like, aka “organotypic”, phenotypes^44^. We hypothesized that we could generate different luminal epithelial cell type compositions in the same cell donor by using a variety of cell culture differentiation media^45–47^, thereby enabling us to compare these tissues to native human *ex vivo* tissues both in terms of structural organization and clearance function. After expanding the airway epithelial cells of multiple donors (**Supplementary Tables S1 & S2**) in a common medium, we differentiated them for 28 days at ALI in one of 5 commonly used cell culture media: BD^48^, mAir^46^, SAGM^™^, PC, or PC-S (see Methods). The tissues differed dramatically in their proportions of luminal ciliated, club, goblet, and hybrid secretory cells depending on culture medium (Fig. 4a; **Supplementary Table S5;** see **Supplementary Figure S9** for another donor). On average, BD, mAir, and SAGM-cultured tissues exhibited relatively low ciliation and contained a substantial proportion of unidentified luminal cells whereas PC and PC-S cultured tissues most closely resembled the average human *ex vivo* composition measured in BG0–6 (Fig. 4b). We used the cellular composition data to organize the different *in vitro* conditions and visualize their relative similarity to different regions along the human airways tree in a physiologically meaningful way. We reasoned that key organizing metrics would include cilia coverage, which is a major determinant of clearance function (Fig. 3e), percentage of MUC5AC+ cells since the presence of mucin 5AC strongly impacts mucus rheology and flow behavior^38^, and the ratio of SCGB1A1+ to MUC5AC+ cell proportions since this ratio changes along the healthy airway tree (Fig. 1d)^25,26^. Here, the definition of SCGB1A1+ and MUC5AC+ cells each includes hybrid MUC5AC+ SCGB1A1+ cells. Mapping the data onto these three dimensions showed that PC-generated cellular compositions were most like human *ex vivo* BG2–6 samples, whereas all other media conditions differed starkly from the human *ex vivo* phenotype (Fig. 4c).

**Figure 5:**
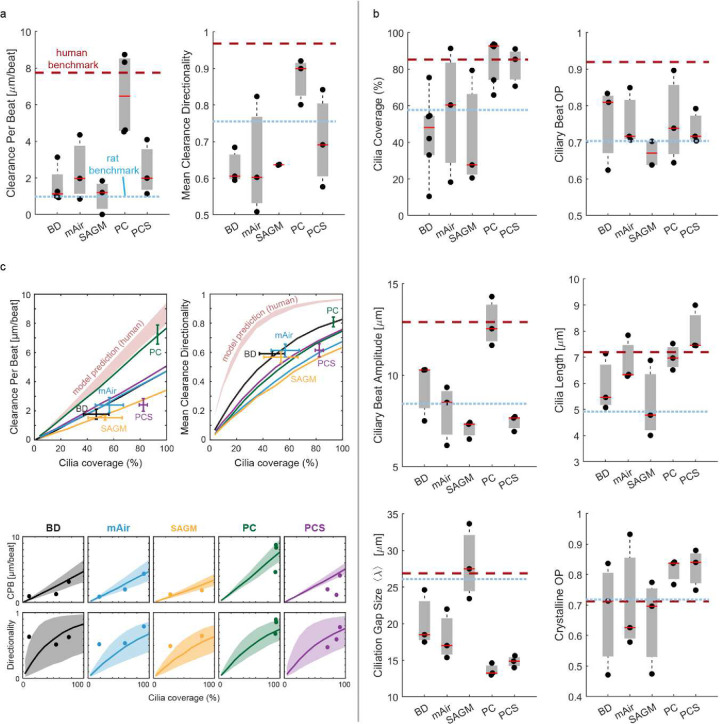
Cell culture media dramatically impact *in vitro* ciliary beat and clearance function. a, Quantitative analysis of particle CPB and directionality in primary human airway epithelial cultures of N=3–4 donors grown in different differentiation media for 28 days at ALI compared to human and rat benchmark data. Each solid dot is the mean value of one donor (across multiple BGs), red line is median of distribution. Dotted lines indicate human and rat benchmark values. b, Quantitative analysis of ciliary beat metrics in airway cells cultured and visualized as in (a). c, Predicted CPB and clearance directionality in *in vitro* cultures (solid lines) compared to predicted human airway performance (red shade). Data points and error bars indicate the particle clearance metrics experimentally measured in each medium (mean ± SEM).

Mirroring these compositional characteristics, only PC-cultured epithelial tissues achieved human *ex vivo*-like particle clearance function with CPB of 6.5 ± 2.3 μm/s and directionality of 0.87 ± 0.04 (Fig. 5a, **Supplementary Video S3**). In contrast, particle clearance in tissues cultured in other media performed below the human *ex vivo* benchmarks and more closely resembled rat *ex vivo* clearance. To understand the mechanistic underpinnings of these results, we assessed all ciliary input metrics (Fig. 5b, **Supplementary Table S4**). Cilia coverage, cilia length, beat amplitude, and ciliation gap size varied the most in response to different differentiation media. On average, BD and SAGM-cultured tissues had shorter cilia than human *ex vivo* samples whereas PC-S cultures had longer cilia, and mAir and PC cultures matched human cilia length (BD: 5.9 ± 1 μm; mAir: 6.8 ± 0.5 μm; SAGM: 5.2 ± 1.5 μm; PC: 7 ± 0.3 μm; PC-S: 8 ± 0.5 μm; human: 7.13 ± 0.5 μm). Notably, only PC-cultured tissues reached human organotypic ciliary beat amplitudes whereas all other culture conditions fell short (BD: 9.4 ± 1 μm; mAir: 8.0 ± 1 μm; SAGM: 7.1 ± 0.3 μm; PC: 12.8 ± 0.8 μm; PC-S: 7.4 ± 0.3 μm; human: 12.9 ± 0.9 μm). Compared to human and rat airways, mean ciliation gap size *λ* was notably smaller in PC and PC-S cultures (BD: 20.2 ± 2.2 μm; mAir: 18.1 ± 2 μm; SAGM: 28.1 ± 3.0 μm; PC: 13.6 ± 0.5 μm; PC-S: 14.8 ± 0.5 μm; human: 24.5 ± 2.9 μm), which is consistent with the comparatively small cell size in PC and PC-S cultured epithelia^49^ (**Supplementary Figure S10).** To assess the functional impact of these differences, we entered the ciliary input metrics of each medium condition into our computational model to predict CPB and particle clearance directionality as a function of cilia coverage (Fig. 5c). Overlaying the measured CPB and directionality values demonstrated a good fit with the model predictions overall, suggesting that our ciliary input metrics alone can be used to predict particle clearance function and explain its divergence from organotypic performance.

### Structural and functional maps enable phenotypic benchmarking.

**Figure 6:**
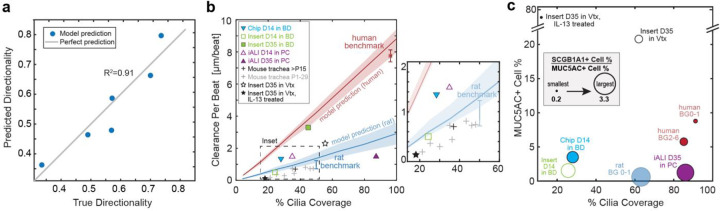
Structural and functional benchmarking of the mucociliary machinery. a, Linear regression model predicting average clearance directionality in human airway epithelia (*in vitro* and *ex vivo*) using as input the average values of MUC5AC+ cell percentage, cilia coverage, and ratio of SCGB1A1+ to MUC5AC+ cell percentages. b, Clearance per beat map comparing different human *in vitro* and rodent *ex vivo* models to human and rat benchmark data. D, day at ALI; P, postnatal day; Vtx, Vertex ALI medium; iALI, human iPSC-derived differentiated airway epithelium. Proof-of-concept data (iALI, Vtx and Vtx +IL13) are from N=1 donor each; other data was sourced from literature, see methods for details. Red line and shaded region: Human model predictions; blue line and shaded region: rat BG0–1 model predictions. c, Cellular composition map comparing different human *in vitro* models to human and rat benchmarks with y-axis: percentage of MUC5AC+ cells; x-axis: cilia coverage, i.e., percentage of ciliated (ATUB+) cells; circle diameter: ratio of SCGB1A1+ to MUC5AC+ cell percentages. Data collected as in (b).

In *in vitro* airway cultures, the physiological development of directional particle clearance depends on the hydrodynamic interaction of dense ciliation with a sufficiently viscous mucus layer; this interaction aligns ciliary beat during differentiation, leading to long-range clearance^50,51^. Mucus viscosity depends on the proportions and abundance of secretory cell types^9,38^; however, tools to assess this relationship remain limited, especially given the difficulty of measuring mucus rheology in miniscule *in vitro* samples^52^. We therefore evaluated whether the key variants in ciliated and secretory cell type composition (Fig. 4c) could be directly predictive of average clearance directionality in the human BG0–6 samples and the *in vitro* cultures. Indeed, a simple linear regression model resulted in a solid prediction of mean clearance directionality in human *in vitro* cultures using as inputs the percentage of ciliated cells, percentage of MUC5AC+ cells, and the ratio of SCGB1A1+ to MUC5AC+ cell proportions (Fig. 6a, coefficient of determination R^2^=0.91). Other input combinations worsened the prediction, including using the percentage of ciliated cells as sole input, or removing it (R^2^=0.59 and R^2^=0.71, respectively), or removing either the percentage of MUC5AC+ cells or the SCGB1A1+ to MUC5AC+ cell ratio (R^2^ =0.29 and R^2^ =0.54, respectively). Hence, combining information on secretory and ciliated cell proportions was most predictive for clearance directionality.

Collectively, our analysis has uncovered the following key findings: (1) structural ciliary metrics, particularly the extent of ciliated cell coverage, are mechanistic predictors of particle clearance function; (2) the selection of cell culture medium significantly influences ciliary metrics and consequently particle clearance function, and (3) the composition of luminal secretory and ciliated cell types may act as a statistical predictor of particle clearance directionality in airway epithelia.

Finally, to demonstrate the broader applicability of our analysis for tissue phenotyping, we leveraged the structural cell-type composition map (Fig. 4c) and the functional CPB-against-ciliation map (Fig. 5c) to assess the mucociliary machinery in additional culture conditions and animal models, where the data was derived from published literature or via proof-of-concept experiments (Fig. 6b–c). The systems evaluated included mature and developing mouse trachea, human airway epithelial cultures derived from human induced pluripotent stem cells, and primary human airway epithelial cultures subjected to asthma-like inflammatory conditions (interleukin-13 (IL-13) treated) or differentiated under mechanical stimulation (Organ-on-Chip models). We found that the pluripotent stem cells-derived airway epithelial tissue differentiated in PC at ALI (iALIs) as described earlier^53^ greatly increased cilia coverage from day 14 and reached nearly human-like levels at day 35 at ALI; however, CPB remained much lower than expected from the extent of cilia coverage, suggesting immature ciliated cell function and organization (Fig. 6b, purple triangles). Secretory cell type composition in iALIs was dominated by SCGB1A1+ cells and showed relatively low levels of MUC5AC+ cells (Fig. 6b, purple circle). The CPB of primary human airway epithelial cultures differentiated in BD was also below the benchmark curve at day 14 of ALI when cultured in conventional static inserts (Fig. 6b, green outlined square) but approached the curve when cultured in continuously perfused Organ-on-Chips (Fig. 6b, inverted cyan triangle). Further, after a prolonged culture time of 35 days, the static insert cultures increased ciliation and reached the CPB benchmark curve, indicating maturation of ciliary beat (Fig. 6b, green solid square). Luminal cell type composition in both insert and chip conditions (Fig. 6c, green and cyan circles) reflected a lack of ciliation compared to the human benchmark; however, the Organ-on-Chip cultures reached nearly organotypic proportions of MUC5AC+ cells^48^. The CPB of primary human airway epithelial cultures cultured in Vertex ALI medium^54^ fell below the benchmark curve at day 35 of ALI (Fig. 6b, black outlined star). Cultures treated with IL-13 for the final 14 days lost cilia coverage and, despite ciliary beat, generated almost no flow at all. The structural map reflects the high proportion of secretory cells expected from culture in Vertex ALI medium^54^ (Fig. 6c, black outlined circle), and IL-13 treatment creates the expected goblet-cell dominated Th2-like asthmatic phenotype^55^ (Fig. 6c, black solid circle). Ciliation and CPB in mouse trachea continue to increase after birth until, at approximately postnatal day (P) 15, they reach their mature performance at 40–45% ciliation^14,56^ (Fig. 6b, light grey crosses and black cross, respectively). The ciliation-dependent increase in CPB parallels the observed and predicted trends in the rat trachea-bronchial airways (Fig. 6b, light blue cross and model prediction), and hence it is possible that mouse and rat trachea share similar ciliary beat properties.

## DISUSSION AND CONCLUSION

We developed a framework to predict how airway morphology influences particle clearance and established benchmarks for comparing experimental airway models against the gold standard, the human airways. Specifically, we discovered that the human large airways exhibit a high degree of ciliation, akin to dogs and pigs^57,58^, while rat airways display a gradual increase starting from 45% ciliation in the trachea. These findings challenge the notion of conserved cilia coverage and clearance function in mouse and human airways^14^. Furthermore, mouse airways diverge from human airways in other relevant aspects, including near 100-fold smaller spatial dimensions, a different branching morphology, and the absence of mucus-producing goblet cells in healthy conditions^59^, similar to our findings in rats. Additionally, the deposition of inhaled particle deposition differs between humans and obligatory nose breathers like rats and mice^60,61^. Consequently, MCC may operate under different constraints in humans and other larger animals compared to rodents. However, these implications remain speculative, as our computational model does not encompass mucus properties, necessitating further investigation.

Our study is first to evaluate culture conditions based on their ability to replicate organotypic characteristics of MCC. The structural and functional maps developed leverage markers commonly measured to visually compare different experimental models with human organotypic benchmarks. While previous studies have assessed the impact of cell culture media on *in vitro* differentiation of respiratory epithelia^46,64,65^, they lacked organotypic benchmarks. Intriguingly, we demonstrated that the composition of secretory cell types may predict clearance directionality, consistent with the need for mucus during differentiation to establish long-range clearance^50^. This relationship provides impetus for future work on mucus microrheology and cilia-mucus interactions.

Our study does have limitations, such as limited availability of healthy human airway samples (13 donors total, as detailed in **Supplementary Table S1**), some of which were extracted from peritumoral tissue. Despite this, the robustly high ciliation levels provide confidence that this is a key characteristic of human airways. Other technical limitations are outlined in the **Supplementary methods.**

In conclusion, our comprehensive structure-function analysis of the mucociliary machinery along the human airway tree provides quantitative benchmarks and visualization tools to assess how human organotypic the mucociliary barrier of experimental models are. The analysis can also probe the effects of maturation, treatments, and diseases on MCC. Our physics-based model linking cilia properties to particle clearance, can aid in estimating the efficacy of treatments aimed at restoring MCC in humans. Importantly, by understanding how tissue-level organization influences ciliated organ function, we shed light on disruptions in mucociliary clearance that play a pivotal role in major respiratory ailments, such as cystic fibrosis, asthma, and chronic obstructive pulmonary disease (COPD). In addition, our approach could be applied to any ciliated organ including function in the fallopian tubes, the ependyma of the brain and the olfactory epithelium.

## METHODS

### Ethical regulations.

All applicable international, national, and/or institutional guidelines for the procurement and use of animal and human cells and tissues were followed. The details of these guidelines and specific protocols are listed in the next section on cell and tissue sourcing.

### Cell and tissue sources.

#### Source of rat airways.

Rat lungs were harvested from freshly euthanized animals used for other research purposes that were performed according to approved animal study protocols. The animals investigated at USC were 3–8 months-old female Wistar-Kyoto rats (Charles River), and the animals investigated at Helmholtz/TUM were 8-week-old female Wistar rats (Charles River) (**Supplementary Table S1**).

#### Source of whole human lung and cells at USC.

Human lung tissue from subjects with no prior history of chronic lung disease was obtained through the International Institute for the Advancement of Medicine (IIAM) with approval from the Institutional Review Board (IRB) of the University of Southern California (USC) (Protocol number: #HS-18–00273). Donor demographics are included in **Supplementary Table S1**. Human trachea-bronchial epithelial cells (HTBECs) were isolated as previously described^66^. Briefly, proximal airways including the trachea, main stem bronchi and 2 further branching generations of the cartilaginous airways were dissected into 1–4 cm^2^ sections and digested in of 0.1% Protease XIV (Sigma #P5147) and 0.001% DNase (Sigma #DN25) (%w/v) in DMEM/F12 (ThermoFisher #11330032) overnight at 4°C. Using a scalpel, epithelial tissue was gently scraped, collected in DMEM/F12, and centrifuged at 400 × g for 5 minutes. After red blood cell lysis in ACK lysis buffer (ThermoFisher #A1049201), epithelial cells were single cell dissociated by incubation with Accutase (Innovative Cell Technologies #AT104) at 37°C for 30 minutes. Cells were then seeded at a density of 30K cells/cm^2^ on PureCol (Advanced Biomatrix #5005) coated tissue culture dishes in airway epithelial growth media (Promocell #C-21160) and passaged at 80% confluence. At each passage cells were seeded at 5×10^3^ cells/cm^2^.

#### Source of human bronchial rings at LUMC.

Bronchial rings were dissected from macroscopically normal lung tissue obtained from patients undergoing resection surgery for lung cancer at the Leiden University Medical Center, the Netherlands (**Supplementary Table S1**). Patients from which this lung tissue was derived were enrolled in the biobank via a no-objection system for coded anonymous further use of such tissue (www.coreon.org). However, since September 1^st^, 2022, patients are enrolled in the biobank using written informed consent in accordance with local regulations from the LUMC biobank with approval by the institutional medical ethical committee (B20.042/Ab/ab and B20.042/Kb/kb). No clinical data from the patients from which the tissues were derived for this study are available.

#### Source of cells at TUM/Helmholtz.

Cells were sourced from commercial suppliers (**Supplementary Table S1**).

#### Source of iPSC-derived cultures.

See section “Data for comparative CPB and cell type composition map.”

#### Source and generation of human primary airway epithelial cell cultures.

Human primary small airway epithelial cells (hSAECs) and trachea-bronchial cells (hTBECs) were obtained from Lifeline Cell Technologies (USA) or via isolation from primary tissue obtained through the International Institute for the Advancement of Medicine (IIAM) with approval from the Institutional Review Board (IRB) of the University of Southern California (USC) (Protocol number: #HS-18–00273). (**Supplementary Table S1**). The first passage cells were expanded in collagen I coated T75 tissue culture flasks and dishes in standard bronchial epithelial cell medium (BEpiCM) (ScienCell) until ~90% confluency. Expanded small airway and bronchial/tracheal cells were seeded on collagen IV (300 μg/mL) coated 12-well 0.4 pore diameter PET Transwell membranes (Corning, 3460) at a density of 150K cells per insert (~135K cells/cm²). The cells from each donor were cultured in BEpiCM supplemented with 1 nM EC23 (Tocris Bioscience) until confluent. Once the tissue was confluent, differentiation was induced by introducing air liquid interface (ALI) via removal of the apical medium (day 0 of ALI culture) and using one of five different differentiation medium in the basal compartment: 1. BD: bronchial epithelial base medium and Dulbecco’s modified eagle medium BEpiCM:DMEM (50:50) with addition of supplements and 50 nM EC23^48^; 2. PC: PneumaCult ALI (STEMCELL Technologies); 3. PC-S: PneumaCult ALI-S (STEMCELL Technologies); 4. SAGM: Small Airway Epithelial Growth Medium (Promocell, C-21170) containing 50 nM EC23; and 5. mAir: a 1:1 mix of Dulbecco’s modified eagle medium and Airway epithelial cell growth medium (AECGM, PromoCell, C-21160) with AECGM supplements and 50 nM EC23 (previously described in ^46^.) The apical surface was washed with phosphate-buffered saline (PBS, no Calcium and Magnesium) at 37 degrees Celsius twice a week to remove excess mucus. Cultures were differentiated until day 28 of ALI.

### Live ciliary beat and particle clearance recordings.

#### Ex vivo samples.

We freshly isolated airway epithelial tissue from respiratory tree branching generation (BG) 0 through BG6 from healthy transplant-rejected human whole lungs, and BG0 through BG5 from healthy rat whole lungs for live measurements. Samples were cut open along the airway tube to reveal the luminal epithelium, submerged in ice-cold HBSS buffer, mounted upside down in a glass bottom dish (ibidi), and gently flattened using a glass cover slip held down with silicone grease at the corners. All live recordings were performed at room temperature, which we found to prolong sample viability compared to higher temperatures. In some samples, ciliary beat was recorded with phase contrast at 100–200 frames per second (fps) using an inverted Leica microscope equipped with a 40× (NA 0.8) objective and a PCO Edge 4.2 high speed camera (Excelitas Technologies) operated with the micromanager plugin (ImageJ). Cilia were also live-stained with fluorescent-dye conjugated wheat germ agglutinin (ThermoFisher; 20 minute incubation in dilution of 1:200)^15^, and 1-μm fluorescent tracer particles were added to the bath, such that live ciliary beat and particle clearance could be recorded in the same field of view for 10 seconds (ciliary beat: 15–33 fps; particle trajectories 8–33 fps) using epifluorescence imaging as previously described^48^. Two to four FOVs with visible CBF were recorded from each sample.

#### In vitro cultures.

*In vitro* cultures were washed for 10 minutes with PBS and recorded at ALI using an inverted Zeiss microscope equipped with a 40× (NA 0.8) phase contrast objective and a temperature-controlled chamber that was preheated to 37°C. Movies were taken at 140 fps using an Orca Flash 4.0 camera (Hamamatsu). To reveal beat kinematics of thicker cultures, samples were mounted upside down and cilia were live-stained with fluorescent-dye conjugated tomato lectin (IVISense^™^ Tomato Lectin 680, Perkin Elmer) by incubating the sample in a 0.25 μM dilution in PBS for 20 minutes. After rinsing, ciliary beat kinematics were recorded at 30 fps using epifluorescence imaging. Clearance was recorded for 10 seconds at 20 fps by adding 1-μm fluorescent tracer particles to the apical surface^48^. Video recordings were made from 2 insert cultures per donor and condition, with at least 8 FOVs per sample.

### Ciliary input metrics.

#### Cilia length in ex vivo samples.

Hematoxylin & eosin (H&E)-stained sections of human bronchial rings and rat airway trachea were imaged using a Zeiss Axioscope 7 fluorescence microscope and a 40× oil DIC objective (NA 1.4). Cilia length data were measured manually using the freehand line tool in the image processing software Fiji ImageJ^67^. Only cilia with visible starting and end point were measured. For each rat donor, on average 15 FOVs were analyzed and on average 10 cilia were measured in each FOV. For each human donor, on average 4 FOVs were analyzed and on average 30 cilia were measured in each FOV.

#### Cilia length in in vitro samples.

The cell layer was dissociated by incubating the cultures for 20 min in prewarmed Accutase (Invitrogen, 00-4555-56) in a conical tube. After 20 min warm culture medium was added at a ratio of 3:1 and the cells were centrifuged at 210× g for 7 min. The resulting pellet was resuspended in 500 μL 4% paraformaldehyde and 10 μL of this suspension was placed onto a glass bottom imaging dish (ibidi,81218–200) and covered with a glass coverslip. Cilia length was measured as above. For each donor, 30 FOVs were analyzed and on average 10 cilia were measured in each FOV.

#### Ciliary beat frequency (CBF).

CBF was measured by applying Fourier spectral analysis to each cilia-associated pixel recorded in high-speed videos as previously described^22^.

#### Ciliary beat order.

We determined ciliary beat orientations using either the ImageJ plugin directional analysis manual tracing of the beat axis from all ciliated cells in at least 3 FOVs, each spanning approximately 200 μm by 200 μm. We derived the director-free ciliary beat order parameter (OP) from the angle distribution as follows: $OP=\sqrt{\langle sin2\theta \rangle^{2}+\langle cos2\theta \rangle^{2}}$, where ⟨⟩ indicates the mean and θ indicates the measured angles.

#### Ciliary beat amplitude.

We measured ciliary beat amplitude by manually tracing the span of the ciliary beat using kymographs of videorecordings^68^ in at least 10 ciliated cells each in 3 FOVs.

#### Cilia coverage, ciliation gap size, and crystalline order.

These metrics were measured from images with IF-stained cilia. Images were thresholded and binarized to reveal the ciliation pattern, and ciliation gap size and crystalline order were estimated as shown previously^14^. Briefly, the 2-point correlation function C(R) was used to measure the probability that two pixels at a certain distance from each other both have a binary intensity level of “1”, i.e., are part of a ciliated cell. For an image with pixel dimensions m × n, the 2-point correlation function is defined as

$$\overset{ˆ}{C}(x,y)=\frac{1}{N}\sum_{i=1}^{m-x}\;\sum_{j=1}^{n-y}\;I(i,j)I(i+x,j+y)$$

where N=(m-x)(n-y)*.* From this function, C(R) is derived by only allowing for coordinate pairs (x,y) that are part of a circle perimeter of radius R (rounded to integer pixel coordinates) and then averaging over the number of pixels in the circle perimeter. The resulting function C(R) is oscillating and its first local maximum reveals λ, the average spacing of two ciliated patches^14^. The crystalline order parameter (COP) describes the degree of variability of the patchiness between multiple FOVs and is derived from the average of λ and its standard deviation std(λ) across FOVs as follows: $COP=\frac{1-\sqrt{2}*sta(\lambda )}{mean(\lambda )}$. Cilia coverage, defined here as percentage of luminal cells that are ciliated, was determined as part of the cell type composition analysis discussed in the associated methods section.

### Particle clearance output metrics.

The displacement and trajectories of fluorescent tracers driven by ciliary beat was automatically measured using the open source ImageJ Trackmate plugin^69^. From these data, two metrics were calculated.

#### Particle clearance directionality.

Particle clearance directionality D(R) was defined in the Eulerian framework. We derived a Eulerian vector field from the particle trajectories by averaging the velocity components (u,v) at each image coordinate (x,y) over all trajectories passing through (x,y) at any time during the recorded video, i.e., $(\overset{‾}{u},\overset{‾}{v}{)}_{\left(x,y\right)}=\frac{1}{N}\sum_{j=1}^{N}\;(u,v{)}_{(x,y),j}$, where N is the total number of trajectories passing through (x,y). This procedure creates a temporally averaged flow vector field, which is useful when tracer particles density is low at any given moment in time. D(R) was defined as the magnitude of the average flow vector divided by the average magnitude of all flow vectors within a square window with side length R, i.e., D(R)=〈v〉∨〈∨v∨〉, where 〈〉 indicates the mean and ∥ indicates the magnitude of each flow vector v. After normalization to mitigate offset, each trace D(R) was fitted with a decaying exponential, $D(R)e^{\frac{-R}{R_{0}}}$, to reveal the correlation length $R_{0}$. To remove ill-fitted curves, we only accepted fits with coefficient of determination above 0.8. We used bootstrapping to estimate the statistics of $R_{0}$ and account for the limited sample size. Since D(R) decays with window size R, we defined its mean value $\left\langle D_{R80}\right\rangle$ by averaging D(R) at R=80μm to assess transport directionality over multiple cell length.

#### Clearance distance per beat.

Clearance per beat (CPB) was defined as the mean speed of particle clearance (in units of μm per second) divided by the ciliary beat frequency (beats per second), resulting in units of μm per beat. This is a measure of the efficacy of each ciliary beat cycle in driving particle clearance. When both speed and CBF data were available for the same FOV, CPB was computed directly from the ratio. When speed and CBF data were recorded separately, their mean values over multiple fields of views were used to compute one single value of CPB from their ratios.

In *ex vivo* samples, the surface topography was often distorted due to elastic recoil after cutting the cartilage rings, leading to contorted tracer trajectories and variable distance between tracer particle and cilia, which impacts apparent clearance speeds (**Supplementary Fig. S6**). To establish benchmarks and to validate the physics-based model, we used the clearance measurements from human and rat samples that we trusted the most, i.e., measurements from entirely flat airway sections where the particles were visibly touching the cilia. For humans, these samples were one recording in donor H44, BG6, and one recording in donor H47, BG2; or rats, these samples were two recordings in donor R43USC, BG0 (see **Supplementary Videos S1 & S2** for one example per species).

### IF staining and imaging.

#### Human and rat airway sections.

Isolated airway tube sections were fixed using a 4% paraformaldehyde (PFA) solution for 3 to 24h depending on tissue thickness, washed with PBS, and stored in PBS at 4 °C until staining. Prior to staining, the diameters of the sections were measured using a ruler. For large airway rings, nearly level sections were dissected at the region of the cartilage to minimize warping of the epithelium after severing the muscle. Small airway samples without cartilage were cut along the tube and flattened out for imaging. Samples were placed into a 96-well plate for staining.

#### Human airway epithelial cell cultures.

After differentiation at ALI, the primary human airway epithelial cultures and iPSC-derived cultures were fixed using incubation with 4% PFA solution for 30 min at RT, then washed again three times with PBS and stored in PBS at 4 °C until staining.

#### IF staining.

Samples were blocked and permeabilized using 0.25% (v/v) Triton-X 100 in PBS with 3% BSA for 60 min at RT, then incubated overnight at 4°C with primary antibodies against secretory proteins MUC5AC and Uteroglobin/ SCGB1A1 diluted in the Triton/BSA buffer (**Supplementary Table S6**). The samples were rinsed three times for 5 min with PBS before incubation with secondary antibodies diluted in Triton/BSA buffer for 1 h at 37 °C, followed by a triple 5 min wash with PBS. Then, the samples were incubated with directly conjugated anti-acetylated-α-tubulin primary antibody (ATUB) to stain cilia, and F-actin stain phalloidin 555 (Invitrogen, A30106) or phalloidin 405 (Invitrogen, A30104) in Triton/BSA buffer for 1 hour at 37 °C, followed by a triple 5 min wash with PBS. The samples were stored at 4 °C until mounting. For mounting, *ex vivo* samples were placed into glass-bottom imaging dishes (ibidi, 81218–200) and covered with SlowFade^™^ Glass Antifade Mountant (Invitrogen, S36917). A round coverslip lined with silicone grease was used to push down and flatten the sections. The *in vitro* samples were mounted by removing the cell culture membrane from the insert using a scalpel and placing the membrane onto a glass slide with the cells facing upwards. The cell were coated with a drop of ProLong^™^ Diamond Antifade Mountant (Invitrogen, P36965) and covered with a round number 1.5 glass coverslip.

#### IF imaging.

Human bronchial ring sections were imaged using a Leica DMi8 microscope equipped with an Andor Dragonfly 200 spinning disk confocal and a 40× water objective (NA 0.80). From every stained ring section, 3 to 8 FOVs with a size of 2048 × 2048 pixels were recorded. Rat samples, other human airway sections, and *in vitro* cultures were imaged using a Leica confocal scanning microscope or a Zeiss Axioscope 7 fluorescence microscope equipped with a 40× oil DIC objective (NA 1.4). Six FOVs with a size of 2048 × 2048 pixels were recorded per sample.

### Cell type composition literature survey.

We reviewed human data from standard histology^25,33,34^ and RNAseq studies^31,32^, as well as rat data from standard histology^25^ (**Supplementary Fig. S4**).

### Cell type composition analysis.

We employed semi-automated image analysis to quantify luminal cell type composition from IF images containing 4 channels (F-Actin, MUC5AC, SCGB1A1, and acetylated α-tubulin (ATUB)) (**Supplementary Fig. S2**). Using ImageJ Fiji^67^, raw image data was converted to 16 or 8-bit tiff images using maximum projection for stacks. As needed, images were cropped to remove edge artifacts and the subtract background function was applied with a rolling ball radius of 1000 pixels. The Fiji plugin Advanced Trainable Weka Segmentation with all default and the Laplacian, Derivatives and Structure training features^70^ was used to segment cell outlines from the F-actin mesh. Channels for cell-type markers MUC5AC, SCGB1A1, and ATUB were filtered and optimized in contrast. After these preprocessing steps, cell type composition analysis was performed by overlaying the cell markers with the cellular outlines using CellProfiler^™71^, providing total cell number and proportions of ATUB+, MUC5AC+, SCGB1A+, and MUC5AC+ SCGB1A1+ cells.

### Airway histology and imaging.

#### Rat trachea.

Tracheas were obtained from wildtype Wistar rats and fixed in 2% PFA at 4°C overnight on an orbital shaker. After washing, samples were embedded into paraffin wax in a lateral (dorso-ventral) orientation. Traditional H&E staining was performed and microscopical images at 400× magnification were taken of all sections, with at least 6 FOVs per trachea.

#### Human bronchial rings.

Bronchial rings were obtained from tumor-free resected lung tissue at the Leiden University Medical Center. The rings were fixed using 4% PFA solution for 24 hours after which the rings were transferred to PBS and stored at 4°C until paraffin embedding at the Department of Pathology at the Leiden University Medical Center. Bronchial ring sections were deparaffinized in xylene and dehydrated in an ethanol gradient. Traditional H&E staining was performed and microscopical images at 400× magnification were taken of all sections, with at least 3 FOVs per sample.

#### Diameter measurements.

Multiple inner diameters were measured and averaged in each cross-section of rat and human airway rings.

### Statistical analysis.

For each metric, the average of the entire FOV was determined, and by averaging this value across all FOVs taken from all samples of the same condition, a single value for each metric was established for each donor and condition. See **Supplementary Table S2** for donor numbers for each condition and measurement. Where noted in the figures, statistical analysis of differences between the median values was performed using the non-parametric Kruskal-Wallis test.

### Linear regression model.

The model was created in MATLAB using the regression learner application.

### Data for comparative CPB and cell type composition map

#### Mouse tracheal CPB.

CPB in developing mouse trachea between postnatal days (P) 0 and 29 (grey crosses in Fig.6b) in were derived from Toskala *et al*^72^*.* CPB in mature mouse trachea at P15 (black cross in Fig.6b) was derived from Ramirez-San Juan *et al*^14^.

#### Airway-on-Chip and insert cultures of primary human airway epithelial cells in BD.

CPB and cell type composition were derived from our previous study^48^.

#### IL-13 treatment of primary human airway epithelial cultures in Vertex ALI medium.

Cells (N=1 donor) were differentiated in Vertex ALI medium^54^ for 21 days at ALI. A chronic airway inflammatory phenotype was induced by treatment with 100 ng/mL of IL-13 (Invitrogen, A42525) for 14 days. Analysis of CPB and cell type composition was conducted at day 35 ALI as described above.

#### Generation and analysis of iPSC-derived respiratory epithelial cultures (iALI cultures).

Differentiation of human iPSCs (hiPSCs) towards respiratory epithelium was performed as described previously^53^. Briefly, hiPSCs from N=1 donor were differentiated to definitive endoderm by using the STEMdiff^™^ Definitive Endoderm Kit (STEMCELL Tech., Vancouver, BC, Canada). Subsequently, cells were dissociated and replated for anterior foregut induction by supplementing basis medium with 10 μM SB431542 (provided by the Institute of Organic Chemistry, Leibniz University, Hannover, Germany), and 3 μM Dorsomorphin (Sigma Aldrich, Saint Louis, MO, USA) for 24 hours, followed by supplementation with 2 μM IWP2 (Tocris, Bristol, UK) and 10 μM SB431542 for another 24 h. For lung lineage specification, basis medium was supplemented with 10 ng/mL BMP4 (R&D Systems, Minneapolis, MN, USA), 10 ng/mL FGF10 (R&D Systems, Minneapolis, MN, USA), and 3 μM Chir99021 (provided by the Institute of Organic Chemistry, Leibniz University, Hannover, Germany) until day 14 of differentiation. NKX2.1 positive lung progenitor cells were enriched by sorting for the cell surface marker carboxypeptidase M (CPM) (FUJIFILM Wako, Cat# 014–27501). To mature lung progenitor cells to ciliated respiratory epithelium, enriched cultures were seeded onto transwells (Greiner Bio-One, Frickenhausen, Germany) and expanded in small airway epithelial cell growth medium (SAECGM; PromoCell, Heidelberg, Germany) supplemented with 1% penicillin/streptomycin (Gibco, Billings, MT, USA), 1 μM A83–01 (Tocris, Bristol, UK), 0.2 μM DMH-1 (Tocris, Bristol, UK) and 5 μM Y-27632 (Tocris, Bristol, UK) for four days. Afterwards, medium was switched to PneumaCult^™^-ALI medium (STEMCELLTech., Vancouver, BC, Canada) and cells were differentiated in air-liquid interface conditions for 28 days before analysis. Analysis of CPB and cell type composition was conducted as described above for primary human airway epithelial cultures.

### Physics-based model.

#### Cell-level ciliary input parameters.

We model the averaged forces generated by all cilia of a multiciliated cell as a single force monopole, located at one cilia length above a no-slip cell surface at z=0, in a semi-infinite domain, based on custom MATLAB (Mathworks) implementation of the regularized Stokeslet algorithm^73^. The regularized Stokeslet’s strength is proportional to ciliary beat amplitude, with its direction corresponding to power stroke direction (**Supplementary Fig. S7A**). While this approach cannot resolve the flow and coordination of individual cilia as in single-cilia models^41,74–77^, it is straightforward to implement, suitable for large number of ciliated cells, and directly takes into account of the wall-screening effects due to finite cilia length comparing to the slip-boundary-velocity approach^14^. We did not consider the effects of double confinement or mucus film geometry discussed in Ramirez-San Juan *et al.* ^14^ because (i) we intend to compare tracer particle motions recorded at the cilia tip and sufficiently far from other confinement boundaries such as coverslip and air-liquid interface; (ii) experimentally, we chose appropriately-sized field-of-views such that no recirculation effects are observable; (iii) all functional measurements were done in washed samples without the presence of mucus.

#### Tissue-level ciliary input parameters.

We derive the ciliated cell distribution based on a cilia coverage percentage and a crystalline order parameter as defined previously^14^, where the order parameter relies on the distribution of wavelength λ between each ciliated patch (**Supplementary Fig. S7A**). Here the mean and standard deviation of λ is determined based on structural measurements. Then ciliated patches are generated based on Gaussian displacement of cells that follow regular crystalline patterns, similar to the procedure reported in Ramirez-San Juan *et al* ^14^. Each ciliated cell assumes a beat direction angle θ sampled from a Von Mises distribution, where its mean is set to be 0 (beating towards x-axis) and its second moment, or the orientation order parameter, is determined based on the measured cilia beat order. In the **Supplementary Methods**, we provide an exact description of how both cilia- and tissue-level parameters are implemented *in silico.*

#### Model output.

The model uses a rectangular grid of 51 × 51 cells, doubly periodic in both x- and y-axis, where x-axis is defined as the clearance direction. Every cell is assumed to have a diameter of 10 μm, with its center slightly perturbed away from the exact grid points in Monte-Carlo simulations; for visual reference, cell boundaries are drawn based on the Voronoi diagram of the perturbed cell center points (**Supplementary Fig. S7B**). We implement the periodic boundary conditions by truncating hydrodynamic interactions further than one periodic image away (>250 μm) from any given point of interest. This introduces only a small error in the flow velocity because the no-slip surface at z = 0 causes a quadratic decay of hydrodynamic interactions. We derive ciliary flow characteristics from the trajectories of simulated tracer particles injected near cilia tip. Tracers are subject to both cilia-driven flow and random fluctuations. All flow experiments were done using 1 μm diameter particles suspended in buffer after the mucus was removed. For predictions associated with *ex vivo* measurements, we assume the random fluctuation is due to only the thermal diffusivity of water at room temperature ( 20°C; D=0.4 μm^2^/s). For predictions related to *in vitro* experiments, we accounted for additional noise, possibly due to immature ciliary beat, and used effective diffusivity scales estimated using particle tracks (**Supplementary Methods; Supplementary Fig. S11**). Time evolution of 500 initially uniformly distributed particles are computed for 500 beat periods, following the Langevin equation $dr/dt=v(r)+(2D{)}^{0.5}\eta (t)$, where r is the particle position, v(r) the cilia-driven flow, and η(t) a standard Wiener process (white noise). Equations are numerically integrated with a Euler-Maruyama scheme, using periodic boundaries in both x- and y-directions. The main quantitative output of our simulation is the clearance per beat (CPB) and clearance directionality (**Supplementary Fig. S7A**).

#### Example flow pattern.

To illustrate how tissue-level parameters affect our model output, we present example case studies (**Supplementary Fig. S7B**). The crystalline order parameter changes how ciliated cells are distributed; however, it does not strongly impact the characteristics of the tracer trajectories. Lowering cilia coverage or orientation order parameters reduces the clearance distance and directionality. In **Supplementary Fig. S8**, we present quantitative results of CPB, and clearance directionality change with (1) cilia length, (2) cilia beat amplitude, (3) cilia beat order and (4) patch heterogeneity.

## Data Availability

All relevant data supporting the key findings of this study are available in the article and its Supplementary information files or from the corresponding authors upon reasonable request. Raw measurement data is available at Figshare: https://doi.org/10.6084/m9.figshare.24989700.
